# Mental health help-seeking behavior among Ukrainian adults during the 2nd year of the 2022 Russian invasion: a cross-sectional study

**DOI:** 10.3389/fpsyg.2026.1793954

**Published:** 2026-03-13

**Authors:** Oleh Lushchak, Mariana Velykodna, Vladyslav Deputatov, Olha Strilbytska

**Affiliations:** 1 Kyiv School of Economics, Kyiv, Ukraine; 2 Biomedical Research Center, Vasyl Stefanyk Carpathian National University, Ivano-Frankivsk, Ukraine; 3 Research and Development University, Ivano-Frankivsk, Ukraine; 4 Department of Biochemistry and Biotechnology, Vasyl Stefanyk Carpathian National University, Ivano-Frankivsk, Ukraine; 5 Department of Psychology, Ukraine Sigmund Freud University, Kyiv, Ukraine; 6 Department of Practical Psychology, Kryvyi Rih State Pedagogical University, Kryvyi Rih, Ukraine

**Keywords:** help-seeking behavior, mental health, Russian invasion, Ukraine, war

## Abstract

**Background:**

The escalation of the Russian war against Ukraine in 2022 resulted in a mental health crisis, requiring prompt and relevant care provision. Numerous services were launched or adjusted to address these issues. However, research reports various obstacles in some individuals, especially from vulnerable groups, to seek help for their mental health condition. This study focuses on the factors associated with mental health help-seeking behavior among Ukrainian adults during the 2nd year of the 2022 Russian invasion.

**Methods:**

An online survey was distributed from 12 October 2023 to 5 February 2024 and collected sociodemographic data, mental health status, and mental health help-seeking behavior during 12 months before survey, along with the Perceived Stress Scale, PTSD-Checklist V, Patient Health Questionnaire, and General Anxiety Disorder inventory.

**Results:**

A total of 9,967 Ukrainian adults (89.8% females) participated in the survey. Receiving mental health care during a year before survey was prevalent in 41.8% of the respondents (18.8% reported regular and 23% occasional visits). It was most significantly associated with a previously diagnosed mental health disorder, meeting PTSD threshold levels, war-forced displacement abroad and within the country, and not being a parent.

**Conclusion:**

These results report the high prevalence of mental health help-seeking behavior in wartime Ukraine, and highlight the role of official diagnosis in it, which appeared to be more influential than having symptoms of depression, anxiety, and perceived stress, except for high PTSD scores. War-forced displacement led people to seek mental health help more often. Parenthood was associated with receiving mental health help less regularly.

## 1 Introduction

The escalation of the Russian war against Ukraine in 2022 resulted in a mental health crisis, requiring prompt and relevant care provision (Humphrey and Forbes-Mewett, 2025). Researchers reported an increase in the prevalence and severity of various mental health issues in Ukrainians in response to this war across the country (Kurapov et al., 2025; Serdiuk et al., 2025; Halabitska et al., 2025) and abroad (Esbit et al., 2025; Guerrero et al., 2023). Burden of perceived stress, anxiety, post-traumatic stress disorder (PTSD), and depression levels were most reported during 2022–2025 as war-related mental health consequences for the Ukrainian population (An et al., 2025; Kurapov et al., 2023; Lushchak et al., 2024; Polyvianaia et al., 2025; Velykodna et al., 2024a).

Numerous services were launched or adjusted to address these issues (Palii et al., 2024; Pinchuk et al., 2025). However, Ukraine-based mental health professionals reported the observed obstacles, specifics, or resistance in some individuals, especially from vulnerable groups, to seek help for their mental health issues in recent years (Dorozhkin, 2024; Puchyna et al., 2025). Identifying at-risk groups and seeking ways of delivering appropriate and effective interventions for specific war-related symptoms, both in clinical and public health settings, is in high demand (Fornaro et al., 2025). Therefore, research focused on the specifics of current mental health help-seeking behavior among various groups of Ukrainians suffering because of the war is of paramount importance.

Generally, only a few studies have explored the specifics of mental health help-seeking behavior in independent pre-war Ukraine, revealing that having a diagnosis, physical health issues, and lower religiosity contributed to the likelihood of seeking care (Jiang et al., 2023; Quirke et al., 2021). After the full-scale Russian invasion of Ukraine, by September 2022, researchers reported low recognition of existing mental health problems and prevalence of seeking relevant support among Ukrainian war refugees within their host countries (Guerrero et al., 2023). In summer 2023, the USA-located Ukrainians tended to seek help only in the case of the high severity and rather long duration of their symptoms due to self-stigma (Artzi-Medvedik et al., 2025). However, other data collected during 2022–2023 showed that Ukrainians who fled abroad were more open to utilizing Ukraine-based hotlines and online support instead of local services due to language issues (Asanov et al., 2023; Palii et al., 2024). Among those who stayed in wartime Ukraine receiving regular mental health care, compared to occasional or none, predicted better wellbeing (Velykodna et al., 2024b).

This study focuses on the factors associated with mental health help-seeking behavior among Ukrainian adults during the 2nd year of the 2022 Russian invasion of Ukraine.

## 2 Materials and methods

### 2.1 Research design

The research was designed as an online survey, which followed the STROBE Checklist for cross-sectional studies (Von Elm et al., 2007). It included a questionnaire on socio-demographic data, mental health status, and mental health help-seeking behavior during the last year, along with a part that assessed self-reported symptoms of perceived stress, anxiety, depression, and PTSD. Help-seeking was defined as contacting a mental health professional including psychologist, psychiatrist, or psychotherapist, as was specified in the survey question.

### 2.2 Variables

The list of the tested variables utilized in this study included: sociodemographic variables (e.g., age, sex, residence, marital and working status, and education levels), a question about mental health help-seeking behavior during the last year, with three options of response (none, occasional, and regular visits), a question on mental health status (previously diagnosed depression, anxiety disorder, PTSD or other disorder) and four diagnostic screening tools: Perceived Stress Scale, PSS-10 (Cohen et al., 1994); PTSD-Checklist V, PCL-5 (Blevins et al., 2015); Patient Health Questionnaire, PHQ-9 (Kroenke et al., 2001); and General Anxiety Disorder inventory, GAD-7 (Spitzer et al., 2006), adapted for Ukrainian population by other authors (Aleksina et al., 2024; Bezsheiko, 2016; Kohut and Chaban, 2025) Probable PTSD status was determined using the DSM-5 symptom cluster scoring rule for the PCL-5. Items scored ≥ 2 (“moderately” or higher) were considered endorsed symptoms. A probable PTSD classification required at least one intrusion symptom (cluster B), one avoidance symptom (cluster C), two negative alterations in cognition and mood symptoms (cluster D), and two arousal/reactivity symptoms (cluster E). This approach follows established recommendations for PCL-5 screening and does not constitute a clinical diagnosis. All instruments demonstrated high to excellent internal consistency in the present sample: PSS-10 (Cronbach’s α = 0.867; McDonald’s ω = 0.867), PCL-5 (α = 0.941; ω = 0.941), PHQ-9 (α = 0.873; ω = 0.874), and GAD-7 (α = 0.891; ω = 0.893). Item-deletion analyses indicated stable item functioning across scales, with no meaningful improvement in reliability following removal of individual items.

### 2.3 Participants

We aimed to recruit adult (above 18 years old) Ukrainians throughout the country, representing different areas of residence, and those who had been forced to flee abroad. The expected sample size was estimated to be a minimum of 1,849 full responses, with a 99% confidence level and a 3% margin of error for the general population of Ukrainians.

### 2.4 Data collection

The survey, constructed via Google Forms, was distributed from 12 October 2023 to 5 February 2024 through several channels, including social media and email lists. The invitation letter consisted of the research description, as well as a link to written consent for participation and the questionnaire with measured variables.

### 2.5 Data analysis

The data analysis utilized the R-based software Jamovi 2.644 and included descriptive statistics, nominal association calculation via Cramer’s V test and multinomial logistic regression.

### 2.6 Ethical approval

The ethical approval for this study was obtained from the Ethical Board of the psychological department of Kryvyi Rih State Pedagogical University in accordance with the Declaration of Helsinki (protocol No 12, 18.05.2023). Every participant signed the consent form.

## 3 Results

### 3.1 Participants

A total of 9,967 Ukrainian adults (89.8% females) aged 18–81 (*M* = 34.8, SD = 9.37, Median = 34) provided full responses to the survey. The full socio-demographic specifics of this sample are provided in Table 1.

**TABLE 1:** Socio-demographic description of the sample.

Item	*M*	Me		SD	Min–max
Age	34.8	34		9.37	18–81
Item			*n*		%
**Gender**					
Male			1,004		10.1
Female			8,950		89.8
Other			13		0.1
**Do you have a child?**					
Yes			4,932		49.5
No			5,035		50.5
**War-forced displacement status**					
Refugees			1,832		18.4
IDPs			873		8.80
NDPs			5,877		59.0
Returnees			1,385		13.9
**Receiving of mental health care**					
Yes, regularly			1,875		18.8
Yes, occasionally			2,289		23.0
No			5,803		58.2
**Education**					
Secondary school			400		4
Secondary specialized education			732		7.3
Higher education			8,229		82.6
Scientific degree			606		6.1
**Type of settlement**					
Village			636		6.4
Urban-type settlement			425		4.3
City			8,906		89.4
**Region of residence (before 2022)**					
Northern			4,061		40.7
Southern			1,191		11.9
Western			1,919		12.3
Eastern			1,163		11.3
Central			1,633		16.4

### 3.2 Mental health help-seeking behavior specifics

Receiving mental health care was prevalent in 41.8% of the respondents (18.8% reported regular and 23% occasional sessions). Table 2 presents the associations between the three types of help-seeking behavior (none, occasional, and regular) and other mental health variables collected for this research.

**TABLE 2:** Associations between help-seeking behavior and mental health variables.

Mental health variables		Receiving of mental health care			Chi-square	Cramer‘s V
		Yes, regularly	Yes, occasionally	No		
General anxiety disorder (GAD-7)	Mild	818 (17.2%)	940 (19.8%)	2,992 (63%)	102*	0.0714
	Moderate	565 (19.7%)	693 (24.2%)	1,608 (56.1%)		
	Severe	492 (20.9%)	656 (27.9%)	1,203 (51.2%)		
Diagnosed anxiety disorder	No	985 (13.3%)	1,454 (19.6%)	4,983 (67.1%)	1,015*	0.319
	Yes	890 (35%)	835 (32.8%)	820 (32.2%)		
PTSD (PCL)	No	869 (16.6%)	1,047 (20%)	3,325 (63.4%)	124*	0.112
	Yes	1,006 (21.3%)	1,242 (26.3%)	2,478 (52.4%)		
Diagnosed PTSD	No	1,590 (17.2%)	2,043 (22.1%)	5,628 (60.8%)	380*	0.195
	Yes	285 (40.4%)	246 (34.8%)	175 (24.8%)		
Depression (PHQ-9)	No depression	147 (13.3%)	199 (18%)	760 (68.7%)	178*	0.945
	Mild depression	437 (16.4%)	533 (20%)	1,693 (63.6%)		
	Moderate depression	505 (19.2%)	591 (22.5%)	1,535 (58.3%)		
	Moderately severe depression	461 (21.1%)	556 (25.4%)	1,171 (53.5%)		
	Severe depression	325 (23.6%)	410 (29.7%)	644 (46.7%)		
Diagnosed depression	No	1,097 (13.9%)	1,603 (20.3%)	5,201 (65.8%)	990*	0.315
	Yes	778 (37.7%)	686 (33.2%)	602 (29.1%)		
Perceived stress-level (PSS)	Mild	43 (13.1%)	57 (17.4%)	228 (69.5%)	114*	0.0757
	Moderate	789 (17%)	936 (20.1%)	2,928 (62.9%)		
	High	1,043 (20.9%)	1,296 (26%)	2,647 (53.1%)		
Other diagnosed mental disorder	No	1,593 (17%)	2,101 (22.4%)	5,666 (60.5%)	422*	0.206
	Yes	282 (46.5%)	188 (31%)	137 (22.6%)		

*< 0.001.

Self-reported mental health behavior during the last year was most significantly associated with a previously diagnosed mental health issue. Among people diagnosed with PTSD, 40.4% reported that they visit psychologists regularly and 34.4% occasionally, compared to 17.2% and 22.1%, respectively, reported by people without this diagnosis. Although the association was statistically significant, the effect size was small to approaching moderate (Cramer’s V = 0.195), indicating limited to modest practical significance. Most of the respondents with diagnosed anxiety disorders received mental health care systematically (35%) or occasionally (32.8%), while only 13.3% and 19.6% without these disorders reported it. Although the association was statistically significant, the effect size was moderate (Cramer’s V = 0.319), indicating meaningful practical significance. Similarly, diagnosed depressive disorders led more people to receive psychological assistance regularly (37.7% compared to 13.9%) or occasionally (33.2% compared to 20.3%). Although the association was statistically significant, the effect size was moderate (Cramer’s V = 0.315), indicating meaningful practical significance. Finally, 46.5% of people with other diagnosed mental health issues visited psychologists regularly, and 31% occasionally, while 17% and 22.4% of the respondents without these issues did so. Although the association was statistically significant, the effect size was small to approaching moderate (Cramer’s V = 0.206), indicating limited to modest practical significance. Among mental health issues assessed via the screening tests (i.e., scales) utilized in this study, only meeting PTSD threshold levels was associated with a tendency to visit mental health professionals regularly (prevalent in 21.3%) or occasionally (26.3%) compared to people with lower PTSD levels (16.6% and 20%, respectively). Although this association reached statistical significance, the effect size was small (Cramer’s V = 0.112), indicating limited practical significance.

Within the group of sociodemographic backgrounds, mental health help-seeking behavior was significantly associated with war-forced displacement experience and not being a parent (Table 3).

**TABLE 3:** Associations between mental health help-seeking behavior and sociodemographic variables.

Sociodemographic variables		Receiving of mental health care			Chi-square	Cramer‘s V
		Yes, regularly	Yes, occasionally	No		
Age group	18–24	212 (17.3%)	267 (21.8%)	747 (60.9%)	194*	0.0987
	25–34	937 (23.3%)	910 (22.7%)	2,169 (54%)		
	35–44	600 (18.6%)	785 (24.3%)	1,842 (57.1%)		
	45–54	115 (9.8%)	262 (22.4%)	794 (67.8%)		
	55+	11 (3.4%)	65 (19.9%)	251 (76.8%)		
Gender	Male	133 (13.2%)	155 (15.4%)	716 (71.3%)	85.3*	0.0654
	Female	1,736 (19.4%)	2,131 (23.8%)	5,083 (56.8%)		
	Other	6 (18.8%)	3 (23.1%)	4 (30.8%)		
Being a parent	No	1,221 (24.3%)	1,113 (22.1%)	2,701 (53.6%)	200*	0.142
	Yes	654 (13.3%)	1,176 (23.8%)	3,102 (62.9%)		
War-forced displacement status	Refugees	505 (27.6%)	538 (29.4%)	789 (43.1%)	298*	0.122
	IDPs	166 (19%)	195 (22.3%)	512 (58.6%)		
	NDPs	898 (15.3%)	1,195 (20.3%)	3,784 (64.4%)		
	Returnees	306 (22.1%)	361 (26.1%)	718 (51.8%)		

*< 0.001.

Ukrainian adults who took refuge abroad were seeking help for their mental health conditions most often: 27.6% reported they did so regularly, and 29.4% – from time to time. Those who returned to Ukraine after taking refuge abroad were the second group, based on their prevalence of visiting mental health professionals regularly (22.1%) or occasionally (26.1%). Among internally displaced people, 19% and 22.3% did so, respectively. And those who were not forced to change their residence visited psychologists most seldom (15.3% regularly and 20.3% occasionally). Although the association was statistically significant, the effect size was small (Cramer’s V = 0.122), indicating limited practical significance. Being a parent contributed significantly to less regular mental health help-seeking behavior, prevalent in 13.3% for regular and 23.8% for occasional visits, compared to 24.3% and 22.1% in the non-parent group. Although the association was statistically significant, the effect size was also small (Cramer’s V = 0.142), indicating limited practical significance.

To account for potential confounding, a multinomial logistic regression was conducted with help-seeking behavior (none, occasional, regular) as the dependent variable and sociodemographic characteristics, displacement status, number of children, prior diagnoses, and symptom measures as covariates. After adjustment, prior clinical diagnoses of anxiety (OR = 2.95, 95% CI [2.56–3.39]), depression (OR = 3.30, 95% CI [2.85–3.83]), and PTSD (OR = 2.09, 95% CI [1.67–2.61]) remained the strongest independent predictors of regular mental health service use (vs. no visits). Higher education was also associated with increased likelihood of regular help-seeking (OR = 2.28, 95% CI [1.65–3.14]), whereas older age (OR = 0.99, 95% CI [0.98–0.99]) and a greater number of children (OR = 0.72, 95% CI [0.66–0.78]) were associated with lower odds of regular care. Symptom severity groups did not remain significant predictors after adjustment. The overall model showed acceptable fit (pseudo R^2^ = 0.099). Full data is provided in Table 4.

**TABLE 4:** Full multinomial logistic regression predicting mental health help-seeking.

Predictor	Occasional vs. none OR (95% CI)	*P*	Regular vs. none OR (95% CI)	*P*
Age	0.997 (0.990–1.003)	0.334	0.986 (0.978–0.994)	<0.001
Gender (1 vs. 0)^a^	1.591 (1.316–1.925)	<0.001	1.507 (1.221–1.860)	<0.001
Gender (3 vs. 0)^a^	2.072 (0.425–10.101)	0.367	3.741 (0.908–15.414)	0.068
Number of children	0.984 (0.918–1.053)	0.637	0.716 (0.658–0.779)	<0.001
Education: secondary specialized	1.054 (0.759–1.463)	0.755	1.126 (0.759–1.670)	0.556
Education: higher	1.479 (1.125–1.942)	0.005	2.276 (1.651–3.140)	<0.001
Education: scientific degree	1.420 (1.005–2.008)	0.047	1.768 (1.162–2.690)	0.008
PTSD diagnosis	1.718 (1.380–2.139)	<0.001	2.089 (1.674–2.606)	<0.001
Anxiety disorder diagnosis	2.192 (1.924–2.497)	<0.001	2.946 (2.564–3.385)	<0.001
Depressive disorder diagnosis	2.227 (1.932–2.567)	<0.001	3.302 (2.848–3.830)	<0.001
Displacement: IDPs	0.578 (0.470–0.710)	<0.001	0.491 (0.392–0.616)	<0.001
Displacement: NDPs	0.525 (0.459–0.600)	<0.001	0.404 (0.349–0.467)	<0.001
Displacement: returnees	0.777 (0.653–0.924)	0.004	0.740 (0.613–0.894)	0.002
PHQ groups	1.065 (0.999–1.135)	0.055	1.043 (0.971–1.121)	0.251
PCL groups	1.137 (1.007–1.284)	0.038	1.053 (0.918–1.207)	0.461
PSS	0.998 (0.986–1.010)	0.726	0.995 (0.982–1.009)	0.499
GAD-7 group	1.017 (0.934–1.107)	0.695	0.883 (0.802–0.972)	0.011

CI, confidence interval.

^a^0, male; 1, female; 3, other.

## 4 Discussion

The obtained results demonstrate the high prevalence of mental health help-seeking behavior in Ukrainians exposed to war, and highlight the role of specific factors in it. Generally, within the surveyed sample, 41.8% of the respondents admitted receiving regular or occasional mental health care over the past year. As the previous data on the prevalence of seeking mental health support in Ukraine is absent, it can only be compared with other countries during the same period. For instance, in 2024–2025, 8.9% of USA-based adults self-reported experiencing a mental health crisis during the past 12 months, and two-thirds of this group reported that they sought help in the mental health care system or beyond (Anderson et al., 2025). In pre-COVID-19 China, 4.5% of citizens sought mental health support at least once in their lifetime (Yin et al., 2019), which shifted to 3.8% of seeking care per year among people traumatized by COVID-19 (Wu et al., 2025). In the Czech Republic, the data collected in 2022 showed that 13.61%–36.4% of adults with various mental health issues admitted to receiving mental health support over the past year (Potočár et al., 2024). In France, population-level data from 2016 to 2022 showed increased mental health care utilization among individuals aged 13–25 years after the COVID-19 pandemic, particularly among females. Outpatient psychiatric consultations rose with relative risks (RR) ranging from 1.08 among 18–25-year-olds to 1.15 among females aged 13–17, hospitalizations for suicide attempts increased with RR of 1.07–1.14, and prescriptions for psychotropic medications also increased, especially among young women (Fond et al., 2025).

Foremost, the revealed high prevalence of mental health help-seeking behavior supports the evidence on the burden of mental health issues and relevant needs for care in Ukrainians in response to war (Kurapov et al., 2023; Lushchak et al., 2024; Polyvianaia et al., 2025). Second, it also indirectly demonstrates some steps forward in addressing the needs of Ukrainians in mental health care services (Dorozhkin, 2024; Pinchuk et al., 2025), including overcoming self-stigma (Artzi-Medvedik et al., 2025) and a move away from the post-communism legacy with the fear of punitive psychiatry (Winkler et al., 2025).

Interestingly, having an official diagnosis appeared to be more influential for seeking care than self-observed symptoms of depression, anxiety, and perceived stress, except for high PTSD scores. This finding aligns with previous research, which revealed no direct correlation between having symptoms and seeking help (Botchway-Commey et al., 2024). It should be noted, that in cross-sectional data an official diagnosis may reflect prior contact with healthcare services rather than directly driving help-seeking. People who have already involved in care are more likely to report a diagnosis, so this association may indicate prior healthcare engagement and access rather than a causal effect. Personal stigma, cultural attitudes to mental health, and previous negative experience of receiving care pose a significant barrier to seeking help (Doll et al., 2021; Evans et al., 2024). In contrast, recognizing a mental health crisis as a feeling of being hardly overwhelmed with a specific mental condition contributes to seeking help from professional care providers, family members, or friends (Anderson et al., 2025). We assume that the diagnosis, as well as a severe condition, could be perceived as a kind of reasonable permission for receiving care.

The exception for people who met the PTSD threshold possibly supports this hypothesis. Recent research involving Ukrainian adults showed that physical health issues and the severity of mental health conditions increased the readiness of people with mental health disorders to seek relevant services for help (Jiang et al., 2023). Moreover, war trauma results in especially severe post-traumatic injuries in Ukrainians compared to other traumatic experiences (Velykodna et al., 2024a). Considering that PTSD symptoms often include intense physical sensations, including sleeping issues, we hypothesize that it makes affected adults more open to seeking and accepting relevant care.

War-forced displacement within the country and abroad, consistent with previous studies, led people to seek mental health help more often. First of all, it reflects the larger prevalence of mental health issues in those Ukrainians who experienced a forcible change of residency (Lushchak et al., 2024). Second, a proliferation of papers reports launching or adjusting mental health services focused on addressing the mental health needs of internally displaced persons (Kokun, 2024; Pinchuk et al., 2025) and war refugees abroad (Asanov et al., 2023; Guerrero et al., 2023; Palii et al., 2024). Therefore, current efforts in supporting forcibly displaced Ukrainians could make this vulnerable group well-equipped with relevant services.

Last but not least, parenthood was associated with receiving mental health help less regularly. Although having a child is associated with higher openness to mental health help-seeking (Gearing et al., 2024), it may pose a barrier to receiving care more systematically. In wartime Ukraine, parents often function as the main or even only mental health care providers for their children traumatized by war (McElroy et al., 2024). As a result, they reported a significant increase in anxiety, depression, a feeling of loneliness, and alcohol use, especially in the case of having a child with emotional or behavioral issues (Hyland et al., 2023). Mental health conditions of war-affected Ukrainian parents make a significant impact on their child (Kapel Lev-ari et al., 2024). Further exploration is required to reveal the gaps in mental health care services for Ukrainian parents and the ways of delivering them adjusted to wartime.

Overall, this research showed the necessity of monitoring the dynamics of mental health help-seeking behavior in Ukrainians and investigating the specific barriers and unmet needs for receiving care. Mental health care institutions and independent services working with Ukrainian adults should focus on overcoming self-stigma and the tendency of Ukrainian adults to underestimate their symptoms. Best practices of providing mental health services in wartime should be explored and disseminated.

## 5 Limitations

This study has several limitations. First, although the sample was large and covered most of the regions throughout Ukraine, it was not well representative in terms of gender specifics, reflecting the specifics of female Ukrainians more, which is common in recent Ukraine-based studies (e.g., Polyvianaia et al., 2025). Gender imbalance likely reflects wartime conditions in Ukraine, where many men of military age are mobilized, serving, injured, captured, or otherwise unavailable for civilian research. Online recruitment through social media and email may also have attracted people with better internet access and greater interest in mental health. As a result, individuals experiencing distress or more willing to seek help may have been more likely to participate, which could be linked to overestimation of symptom levels and help-seeking. Second, the sample included a general, i.e., non-clinical, population. Therefore, the variable of having an official diagnosis was self-reported and could differ from an objective. The relationship between diagnosis and help-seeking may also be bidirectional, as service contact increases the likelihood of diagnosis and vice versa. Finally, recent research demonstrated response process validity with PHQ-9 as a diagnostic tool, in which respondents often understand the instructions incorrectly (Panayiotou et al., 2025).

## Data Availability

The raw data supporting the conclusions of this article will be made available by the authors, without undue reservation.
